# MKEM: a Multi-level Knowledge Emergence Model for mining undiscovered public knowledge

**DOI:** 10.1186/1471-2105-11-S2-S3

**Published:** 2010-04-16

**Authors:** Ali Z Ijaz, Min Song, Doheon Lee

**Affiliations:** 1 Department of Bio and Brain Engineering, KAIST, South Korea; 2 Information Systems, New Jersey Institute of Technology, USA

## Abstract

**Background:**

Since Swanson proposed the Undiscovered Public Knowledge (UPK) model, there have been many approaches to uncover UPK by mining the biomedical literature. These earlier works, however, required substantial manual intervention to reduce the number of possible connections and are mainly applied to disease-effect relation. With the advancement in biomedical science, it has become imperative to extract and combine information from multiple disjoint researches, studies and articles to infer new hypotheses and expand knowledge.

**Methods:**

We propose MKEM, a Multi-level Knowledge Emergence Model, to discover implicit relationships using Natural Language Processing techniques such as Link Grammar and Ontologies such as Unified Medical Language System (UMLS) MetaMap. The contribution of MKEM is as follows: First, we propose a flexible knowledge emergence model to extract implicit relationships across different levels such as molecular level for gene and protein and Phenomic level for disease and treatment. Second, we employ MetaMap for tagging biological concepts. Third, we provide an empirical and systematic approach to discover novel relationships.

**Results:**

We applied our system on 5000 abstracts downloaded from PubMed database. We performed the performance evaluation as a gold standard is not yet available. Our system performed with a good precision and recall and we generated 24 hypotheses.

**Conclusions:**

Our experiments show that MKEM is a powerful tool to discover hidden relationships residing in extracted entities that were represented by our Substance-Effect-Process-Disease-Body Part (SEPDB) model.

## Background

The advent of high-throughput methods and sheer volume of medical publications covering various diseases, mining Undiscovered Public Knowledge (UPK) from these resources is a daunting challenge. The concept of UPK was introduced by Swanson in discovering Raynaud disease and fish-oil relation in 1986 [1]. Swanson defined UPK is public and yet undiscovered in two complementary and non-interactive literature sets of articles (independently created fragments of knowledge), when they are considered together, can reveal useful information of scientific interest not apparent in either of the two sets alone [1,2].

Swanson semi-automatically analyzed scientific articles by using exploratory methods so as to mine for cause-effect relations. He showed that chains of causal implication within the medical literature can lead to hypothesis for cause of rare diseases, some of which may receive scientific supporting evidence.

**Figure 1:**
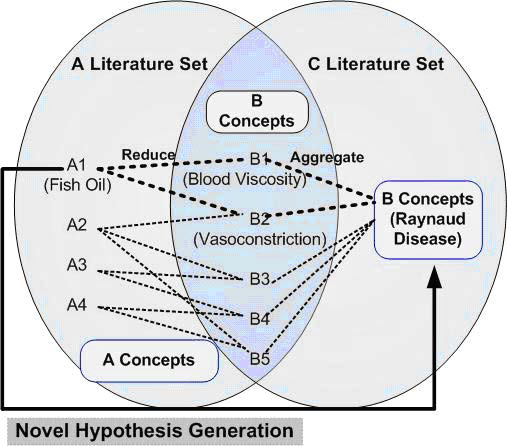
Swanson’s UPK model – the connection of fish oils and Raynaud disease

The underlying discovery method is based on the following principle: some links between two complementary passages of natural language texts can be largely a matter of form “A cause B” (association AB) and “B causes C” (association BC) (See Figure 1). From this, it can be seen that they are linked by B irrespective of the meaning of A, B, or C. However, perhaps nothing at all has been published concerning a possible connection between A and C, even though such link if validated would be of scientific interest. This allowed for the generation of several hypotheses such as “Fish's oil can be used for treatment of Raynaud's Disease” [3].

One major issue with the Swanson’s approach is that it requires the labor intensive work of a domain expert in the process of screening out the intermediate concepts (the “B” concepts) [4]. To overcome this issue, several approaches [5-8] have been proposed to automate the Swanson’s UDK method. Even though these approaches have successfully replicated the Raynaud disease/fish-oil and migraine/magnesium discovery, it still requires substantial manual intervention to reduce the number of possible connections. In addition, existing approaches do not cover hidden relations resided at the molecular level.

Several techniques have been proposed to automate the Swanson’s UDK model. Early studies on the UDK model applied advanced Information Retrieval techniques such as Latent Semantic Indexing (LSI) and TF*IDF to find candidate intermediate concepts on top of ranking term lists [9-11]. They easily identified high ranking intermediate terms of interest. However, applying the same statistics to the intermediate literatures, the already known (by Swanson’s work) target terms such as Fish Oil could not be found directly in higher ranks. Apart from statistical approaches to the UDK model, rigorous attempts were made to integrate external knowledge in ontologies into the discovery process in the UDK model [12,13][40]. Srinivasan [14] viewed Swanson’s method as two dimensions. The first dimension is about identifying relevant concepts for a given concept. The second dimension is about exploring the specific relationships between concepts. However, Srinivasan [14] dealt only with the first dimension. The key point of her approach is that MeSH terms are grouped into the semantic types of UMLS to which they belong. However, only a small number (8 out of 134) of semantic types are considered since the author believes those semantic types are relevant to B and A concepts. For each semantic type, MeSH terms that belong to the semantic type are ranked based on the modified TF*IDF. There are some limitations in their method. First, the author used manually-generated semantic types for filtering. Second, the author applied the same semantic types to both A and B terms. Because the roles of A and B terms for C term are different, different semantic types should be applied.

Hristovski, et al. [12] used the MeSH (Medical Subject Headings) descriptors as features and employed association rule algorithms to find the co-occurrence of the words. Their technique first found every intermediate B concepts related to the concept C and then all A concepts related to B concepts were selected by searching Pubmed. Since each concept can have one or many relationships with other concepts, the size of B→C and A→B combinations can be extremely large. In order to deal with this combinatorial problem, the algorithm incorporates filtering and ordering capabilities. Hu et al. [4] utilizes the semantic types and semantic relationships of the biomedical concepts through Unified Medical Language System (UMLS). Their system identifies the relevant concepts collected from Medline and generates the novel hypothesis between these concepts. Pratt and Yetisgen-Yildiz [6] used UMLS concepts instead of MeSH terms and limited the search space to the document titles as a starting concept which is similar to Swanson’s method to reduce the number of terms (B concepts and A concepts). They also reduced the number of terms/concepts by classifying terms into three categories: “too general”, “too closely related to the starting concept”, and “meaningless”. With the qualified and grouped UMLS concepts, they used the well-known Apriori algorithm [15] to find correlations among the concepts. By concept grouping they were able to discover Swanson’s migraine-magnesium implicit connection. However, their technique required strong domain knowledge in selecting semantic types for A and B concepts.

Atkinson and Rivas [13] used NLP techniques, in a similar manner, to extract cause and effect relationships related to diseases from biomedical text and infer new hypothesis from the information extracted. The system used the concept types of “substances”, “symptoms” which represented symptoms of a disease, “diseases” and “body parts”. While the system aims to infer emergent knowledge, it was limited in scope. The system was extracting at the physiological level. Humanly created discovery patterns were defined by biomedical experts using the training corpus. And additionally, manual extraction patterns were also used to create a symptom list. Validation for information extraction part of the system was not performed and instead, some of the transitive relationships that the system developed were given to human experts for evaluation, thereby representing a weak evaluation of the system. Inferring indirect relationships from biomedical text is generally considered challenging however it is also potentially more rewarding. As the literature is so vast that each researcher can only read a small subset, it might be that no person is aware of all the facts that are required to make a logical indirect inference of related facts. These research works have made significant progress on Swanson’s method. However, none of the approaches considers the various different biological entities such as body parts, DNA, and RNA other than disease and cure.

In addition, several studies have identified and extracted biological information from unstructured biological corpus by building on the UMLS knowledge sources [16-18]. SemRep is an outcome of such studies that serves as a general knowledge-based semantic interpreter and a host of tools to extract important knowledge contained within large text corpus.

The goal of this paper is to propose a novel and fully automated approach to mine undiscovered public knowledge from biomedical literature and develop a flexible discovery model that can be applied to various different biological entities.

The contribution of this paper is 1) proposing a flexible knowledge emergence model to extract implicit relationships across different levels such as molecular level for gene and protein and Phenomic level for disease and treatment, 2) employing MetaMap for tagging biological concepts, and 3) providing an empirical and systematic approach to discover novel relationships based on similarity between substances/drugs thereby providing a measure to gauge the newly formed relationships. Our MKEM model is not only differentiated from but also a sophisticated model than Swanson’s UPK model as Swanson’s method does not perform any similarity measure between substances/drugs. Our approach requires presence of multiple extracted relationships containing similar substances before we could aim to produce new hypotheses. In addition, Swanson’s method does not provide any measure to gauge the newly formed hypotheses.

We fully automate the discovery process in the UDK model based on the semantic knowledge about the medical concepts and their relationships. We also propose similarity measure to prune irrelevant medical concepts and bogus or non-interesting relationships among the medical concepts. Our use of an intermediate set of automated identified semantic types helps to manage the sizable branching factor.

## Methods

**Figure 2:**
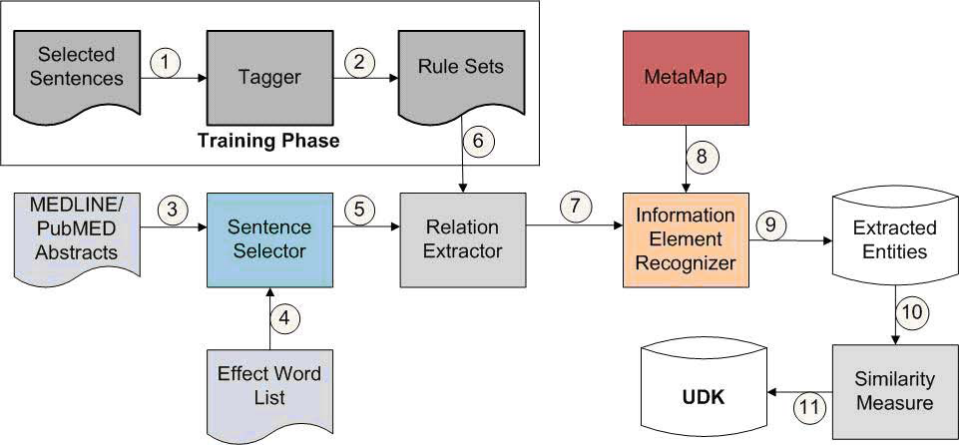
Data flow of MKEM

In this section, we describe our approach for knowledge emergence. First we give an overview of our system (see Figure 2) and describe how it works in steps. Second we introduce the SEPDB information model defining entities extracted from the biomedical text. The learning process for relation rules is described under the “Learning a rule set” section. Third we describe the extraction process and the concept of similarity measure.

**INPUT:** MEDLINE Abstracts

**OUTPUT:** New Relationships

**STEP 1:** Selected sentences are provided to the tagger for concept tagging. These sentences contained the relationship on which our SEPDB model is based. About 150 sentences were selected by the authors from biomedical texts to use for rule generation, from which 100 rules were generated.

**STEP 2:** Rule sets are generated by the tagger for extraction purposes. Step 1 and 2 is to learn rules. We select candidate sentences that contain terms representing relationships based on the SEPDB model. These sentences are then fed to the “tagger” for tagging important concepts. The concepts are based on the SEPDB information model of the system. This leads to rule creation where each rule defines a path between different concepts. The user “tags” words of interest in the tagger. This provides an intuitive as well as a faster way of creating rule set.

**STEP 3:** MEDLINE abstracts are downloaded and given to the sentence selector.

**STEP 4:** Effect Word List is fed into the sentence selector. These words are searched in the new sentence and represent the main connector in our relationships.

**STEP 5:** Any matched sentence containing the effect keyword is handed over to the relation extractor which performs the extraction process.

**STEP 6:** For the sentence containing the effect keyword, the rules related to the keyword are read by the relation extractor.

**STEP 7:** The extracted data is given to the Information Element Recognizer for named entity recognition.

**STEP 8:** MetaMap is employed as a NER (Named Entity Recognizer) engine and tags the information provided.

**STEP 9:** After the NER process, a set of biological entities is extracted by the system for further analysis. Step 3 to Step 9 is to extract entities and their relation. Sentences that contain certain effect words are extracted from MEDLINE abstracts. These sentences are then parsed by the link grammar parser. Rules that were created by the tagger are applied to extract the relevant information from the sentences. Additionally, MetaMap is used as a NER for identification purpose as well as sorting of the concepts if required. MetaMap used UMLS Metathesaurus which provides better coverage of the concepts involved as well as uses standard semantic types.

**STEP 10:** The extracted information is utilized for the similarity measure.

**STEP 11:** The application of similarity measure produces new relationships and it is given as output. Step 10 and 11 is for hypothesis generation. Similarity measure is used for formation of new relationships/hypothesis. This similarity measure is used to gauge the similarity between substances, the more similar the substances, the more possible the newly created relationships.

### SEPDB information model

**Figure 3:**
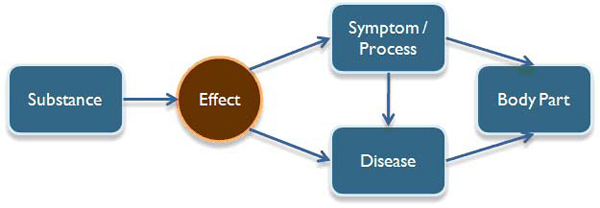
SEPDB information model

Our information model is termed as SEPDB (Figure 3), which stands for **S**ubstance, **E**ffects, **P**rocesses, **D**isease, **B**ody Part. Each of this is a concept extracted from natural language text. We include low-level processes that a substance may affect or that may occur in a disease or body part. This provides us with a better insight as to function of a drug or a substance and what low-level processes it is affecting. In addition, our system also extracts information that contains processes that occur in a specific body part e.g. a cell.

“Effect” node acts as the main node that connects the whole relation. Substance node is directly related to the effect node as an effect is an attribute of a substance. Such attribute can influence a process or a disease. Lastly process or disease can occur in a body part. The Effect keywords and their types are given in Table 1. These effect words are searched in the sentences for learning or extraction purposes. Their types describe the general action taken by the substance. They act as a connector between the substance concept and either the disease or the process concept in the extracted relationships.

**Table 1:** Effect list and types

Effect	Type
Induce	Increase
Contribute	Increase
Reduce	Reduction
Increase	Increase
Resistant	Reduction

### Learning a rule set

To create a rule set, candidate sentences are selected to represent the relationship identified by our data model. The sentence is fed to the Tagger that parses the sentence using Link Grammar Parser and displays the parsed sentence. The relevant concepts such as Disease, Substances, Processes, Effects, Body parts are then tagged visually.

We can think of a parsed sentence as a graph where words are vertices and links are edges. Therefore a rule is the shortest path between an effect word or a keyword and a concept. This connection is stored as a rule. Hence, a rule is created by first selecting a word as an effect and traversing the graph to the other tagged concepts.

**Figure 4:**
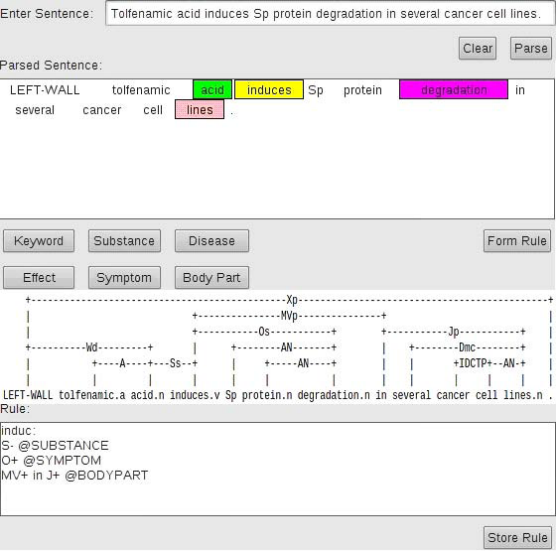
Rule creation

 The link labels are stored in their reduced form, storing information only about the primary link. Directionality information is also stored by using “+” and “-” signs that represents search directions for right and left respectively. Intermediate words are termed as nodes and a rule can have any number of nodes. An example provided in Figure 4 illustrates the aforementioned concept.

The words are stemmed by Porter Stemming algorithm [19] to solve the problem of inflection. For a rule to be satisfied, the input sentence must contain words and links defined in the rule. In other words, with a sentence being a graph in Link Grammar, a rule is a route. Rule satisfaction occurs when the portion of the parsed sentence, starting from the effect word, contains the links and words defined in the rule and the route completely traversed.

An example of how a rule set is created using the proposed technique is as follow (Figure 4):

1) a sentence is selected and parsed by the Link Grammar Tagger. As illustrated in Figure 4, a sentence is entered into the tagger to be parsed, and the user tags the concepts. The system displays the word linkages and the resultant rule set.

2) The user tags the concept of importance and the effect word in the sentence.

3) The program also displays the sentence linkage provided by the Link Grammar Parser.

4) Finally, when tagging is complete, a rule set is formed and displayed by the system. It can then be stored in the rule set file.

 As an example (Figure 4), one of the rules generated by the tagger is “S- @ SUBSTANCE”. Beginning from the effect word, in this case “induces”, we move towards the left, following the S link. On the left, we find the word “acid”, which is the substance inducing an effect. The system automatically expands the name of entities. Hence the system follows such rules to traverse the generated graph to extract the entities of concern.

As shown, a rule may have the following place holders for concepts:

@SUBSTANCE : Representing substances, drugs and related concepts.

@SYMPTOM: Representing symptoms and processes.

@DISEASE: Representing diseases.

@BODYPART: Representing body parts.

Effect words do not have a placeholder as they are represented in the rule set. In this manner, using different sentences, a rule book is created and used by the extraction system for information extraction purposes. As is seen in the newly formed rules, directionality information is shown with “+” and “-” signs. The information is extracted from natural language text when these rules are satisfied by the sentences.

### Extraction

MEDLINE abstracts in XML format are fed to the Sentence Selector. The “Sentence Selector” extracts sentences from abstracts and for each sentence; the words are stemmed and checked against a list of effect words. If a match occurs, the sentence is passed to the “Relation Extractor” module and rules related to the effect word matched are applied on the target sentence and relevant information is extracted. After extraction of data, the output is fed to the “Information Element Recognizer” process to the followings: 1) Removes any unknown word from the dataset: This reduces false positives. For the words that do not occur in MetaMap, they are removed.

2) Correctly sorts the identified word and allocates it to its correct position as one of the four concepts: It is possible that a disease is incorrectly extracted as being a symptom based on the rule set being used. In order to resolve this incorrect assignment, MetaMap is used to properly shift the concept into its correct place.

Extracted data is mapped onto the SEPDB information model, and the relationships conforming to the model are then stored as output. After the data sets have been created, they are then used to infer new knowledge by combining multiple pieces of information using similarity measure.

### Similarity measure

To discover novel relationships, we propose a semantic similarity measure that calculates resemblance between substances. The assumption for this measure is that if the substances shares similar properties with each other, novel connections exist among related concepts to the substances... The similarity measure is also used to rank the newly formed relationships.

The similarity measure comprises of four units.

• MetaMap Semantic Type

• Structural Similarity

• Atomic Count

• XLogP

MetaMap semantic type represents the UMLS semantic type assigned to the substance. As MetaMap categorizes the substances into predefined UMLS semantic types, it assumes that substances under same category may perform similar actions.

Structural similarity is calculated using the SMSD (Small Molecule Subgraph Detector) system [20]. Structural similarity plays a very important role in medicinal sciences. Substances having highly similar structures are more likely to exhibit the same actions.

Atomic count and XLogP values are taken from the chemDB database. Atomic count defines the enumeration of constituent atoms of the chemical under consideration. For small molecules like drugs, atomic count is considered valuable for similarity purposes.

In the fields of organic and medicinal chemistry, a partition (P) coefficient is the ratio of concentrations of a compound in two phases of a mixture of two immiscible solvents at equilibrium (Water-Octanol). XLogP represents its logarithmic form. Hence this describes whether a substance would dissolve more in a water based medium like blood or hydrophobic medium like lipid bilayers of cells. Partition coefficients are useful in estimating distribution of drugs within the body. Therefore, for similar drugs, their dissolution in hydrophobic or hydrophilic medium should be same or similar. Comparative values for the similarity measures are shown in Table 2. The sum of these values is used for ranking of newly created relationships.

**Table 2:** Similarity measure comparative values

Comparative Values
**MetaMap Type**	**Structural**	**Atomic Count**	**XLogP**

0: Not Similar	0: Not Similar	0: Not Similar	0: Not Similar
1: Similar	0.5: Substructure 1: Similar	1: Similar	0.5: Somewhat similar (1 < diff <0.5) 1:Similar

The similarity measure can have a maximum value of 4. We selected a threshold value of 2 for the created hypotheses. Therefore relationships having a score greater than or equal to the threshold are considered and all others are dropped. Additionally, the score values are also used for sorting the newly formed relationships.

### Similarity measure working scenario

The following scenario is given to help understanding of how similarity measure is calculated and applied. For two substances, “Cordycepin” and “Fludarabine”, we check the semantic type assigned by MetaMap for each substance. “Pharmacologic Substance” is assigned to both of them by MetaMap. Next we calculate structural similarity of these substances. For that purpose we find out their structural formula or SMILES (simplified molecular input line entry specification) value available at chemDB database. SMILES is a specification for unambiguously describing the structure of chemical molecules. After acquiring the SMILES values, we supply the SMSD system the values to calculate the structural similarity, which in this case comes out as 0.9. This denotes the two chemical being structurally very similar.

Atomic values and XLogP values can be acquired from the chemDB database. Entering either the chemical name or SMILES formula gives us the information on the chemical under question, including the atomic values and XLogP. Cordycepin has the atomic value C_10_H_13_N_5_O_3_ and Fludarabine has C_10_H_12_FN_5_O_4_. XLogP values are -1.25 and -1.38 respectively. Lastly, when all of these four values are acquired, we use our comparative values table to calculate the total similarity measure (Table 3)

**Table 3:** Calculated similarity measure for two substances

	Cordycepin	Fludarabine	Similarity
**MetaMap Semantic Type**	Pharmacologic Substance	Pharmacologic Substance	1
**Structural Similarity**	0.9		1
**Atomic Count**	C_10_H_13_N_5_O_3_	C_10_H_12_FN_5_O_4_	1
**XLogP**	-1.25	-1.38	1

**Total**			**4**

With a high similarity value, we can assume that both substances perform similar action and therefore we can make new relationships from combining extracted information containing them.

## Results

### Performance analysis

**Figure 5:**
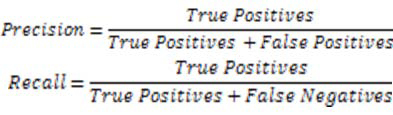
Formulae

For the MEDLINE abstracts, we searched “Cancer” on PubMED database and downloaded 5000 abstracts in XML format. Total 410 relationships were extracted from the downloaded dataset Statistics of the extracted entities is shown in Table 4. As gold standard is not available to evaluate the performance of our system, we conducted the performance analysis of our information extraction module by randomly selecting 98 sentences containing relationships and calculated the precision and recall using the formulae given in Figure 5.

**Table 4:** Extracted entities count

Entity Type	# of extracted entities
Substances	410
Processes	357
Diseases	44
Body Parts	82

Precision means the proportion of relevant documents from all the results retrieved, Recall refers to the proportion of retrieved documents, out of all relevant results available. Results of the analysis are shown in Table 5. True Positives are those relationships that were correctly extracted from the dataset. False Positives are incorrectly extracted relationships whereas false negatives are those relationships that were present in the text but were not extracted. We calculated the correct and incorrect relationships. Considering the complex relationship structure represented by our SEPDB model, the performance of our system looks promising. With 75% in precision, the system extracted correct information with very few false data considered as true. Concerning recall, the system was able to extract 56% of the information that was present in the input text.

**Table 5:** System performance analysis

System Performance
Accuracy	Precision	Recall
56%	75%	56%

The experiment of hypothesis generation was carried out for only those substances that exhibited similarity to each other. In total we were able to infer 24 hypotheses from the extracted data set. Table 6 shows selected four examples of extracted data set along with the raw sentences. This represents the extraction of information from sentences. After the extraction process, we apply the similarity measure to create new hypotheses.

**Table 6:** Sample dataset with raw sentences and extracted information

PubMed ID: 19264955 Sentence: These results show that **fisetin induces apoptosis** in **HCT-116 cells** via the activation of the death receptor- and mitochondrial-dependent pathway and subsequent activation of the caspase cascade.				
Substance	Effect Type	Process	Disease	Body Part

fisetin	increase	apoptosis	N/A	HCT-116 Cells

PubMed ID: 19262372 Sentence: **Docetaxel** was a more potent **inducer** of **apoptosis** than SN-38, but simultaneous treatment with docetaxel+SN-38 decreased the proportion of apoptotic cells to the same level observed after exposure to SN-38 alone.				

Substance	Effect Type	Process	Disease	Body Part

Docetaxel	increase	apoptosis	N/A	N/A

PubMed ID: 18070986 In this study, we show that **Wogonin**, derived from the traditional Chinese medicine Huang-Qin (Scutellaria baicalensis Georgi), **induces apoptosis** in **malignant T cells** in vitro and suppresses growth of human T-cell leukemia xenografts in vivo.				

Substance	Effect Type	Process	Disease	Body Part

Wogonin	increase	Apoptosis	N/A	malignant T Cells.

PubMed ID: 19258429 Sentence: **Tolfenamic acid induces Sp protein degradation** in several **cancer cell lines**.				

Substance	Effect Type	Process	Disease	Body Part

Tolfenamic Acid	increase	Sp protein degradation	N/A	Cancer cell lines

### Hypothesis generation example

Two relationships taken from the extracted data set are given in Table 7. From these extracted relationships, the names of the substances are extracted and similarity measure is calculated as showed in Table 8.

**Table 7:** Example relationships

Substance	Effect Type	Process	Disease	Body Part
Wogonin	Increase	Apoptosis	N/A	Malignant T Cells
Fisetin	Increase	Apoptosis	N/A	HCT-116 Cells

**Table 8:** Similarity measure

	Wogonin	Fisetin	Similarity
MetaMap Type	Organic Chemical		1
Structural Similarity	0.75		1
Atomic Count	C_16_H_12_O_5_	C_15_H_10_O_6_	1
XLogP	2.74	2.77	1

Total			4

The two substances, “Wogonin” and “Fisetin”, belong to the same MetaMap semantic type. Using SMSD, their structural similarity value comes up as 0.75. From chemDB, their atomic count and XLogP values are very similar. Therefore, based on the comparative values, the total similarity score comes up as 4.

As the substances are similar to each other, we proceed to create new relationships. In this case, both processes are identical except for difference in the body part in which these processes occur. Therefore we create new relationships with the substance, effect type and process taken from one relationship and the body part taken from the other. The newly created relationships are shown in Table 9.

**Table 9:** Newly formed relationships

Substance	Effect Type	Process	Disease	Body Part	Score
Wogonin	Increase	Apoptosis	N/A	HCT-116 Cells	4
Fisetin	Increase	Apoptosis	N/A	Malignant T-Cells	4

In Table 9, the first relationship state that “Woginin” can induce “Apoptosis” in **“HCT-116 Cells”.** Comparing that with Table 7, the original “Woginin” relationship had **“Malignant T Cells”** as the body part. In essence, with the process “Apoptosis” being the same for both drugs, the body parts in which the process occurred were switched and the two new relationships were created.

In our approach, the generation of new hypothesis does not require any human input. The initial relationship information is extracted automatically from biomedical text (Table 7) and similarity measure calculation (Table 8) is performed using chemical information from freely available chemical databases. Next, the hypothesis generation utilizes this information to form new relationships from biomedical text (Table 9). Application of similarity measure to extracted relationships produced new hypotheses and a sample data set of such relationships is given in Table 10.

**Table 10:** Sample of newly formed relationships and associated scores

Substance	Effect Type	Process	Disease	Body Part	Score
Wogonin	Increase	Apoptosis	N/A	HCT-116 Cells	4
Fisetin	Increase	Apoptosis	N/A	Malignant T Cells	4
Docetaxel	Increase	mRNA expression of IL-1	N/A	N/A	3.5
Genistein	Increase	Apoptosis	N/A	HCT-116 Cells	2
Fisetin	Increase	Apoptosis	N/A	Tumor Cells	2

## Discussion

Given that the main goal of our approach is for automatic hypothesis generation, we attempt to verify validity of the discovered novel connections discovered by searching for such findings published in the scientific literature. As listed below, we have found two scientific articles that support the existence of the novel relationships discovered by our approach.

Taking example of the first generated relationship in Row 1 of Table 10, Wogonin increases apoptosis in HCT-116 cells. HCT-116 cells represent Human colon cancer cells. This newly formed relationship is supported by the research article by Dae-Hee Lee et al. [21].

Row 4 states that Genistein can induce apoptosis in HCT-116 cells. By searching PudMed, we came across a research article by Mao Li et. al. [22] In this article, they state that Genistein has chemopreventive effects in several human malignancies, including colon cancer and induces apoptosis in a variety of human cancer cell lines. For the rest of discovered relationships, literature search did not find any relevant research articles. It may be a good research topic to investigate whether there exists such a novel connection among them.

## Conclusions

We proposed a new system that extracts relationships from biomedical text and infers new information. This system can be used for knowledge emergence tasks as it combines information from multiple disjoint sets of information (research articles etc) and provides novel hypotheses that may either be correct or would lead to a promising research direction. The system was applied on SEPDB-driven relationships and we achieved good extraction accuracy from natural language text. In addition, using the similarity measure concept, we were also able to infer new relationships, showing that our system is able to perform its task well.

There are multiple options for further improvements. First, we plan to replace MetaMap with machine learning techniques for Named Entity Recognition. This should improve the results as more entities such as drugs and processes are recognized by the system. Second, we will extract anaphoric relationships that exist within a sentence to increase the performance. In addition, we are improving Link Grammar lexicon to reduce incomplete or incorrect word linkage considerably and in consequence providing better parsing results. Last, we plan to carry out rule generalization to reduce the rule sets and provide better coverage of extracting possible relationship from the text.

## Competing interests

The authors have no competing interests to this research.

## Authors' contributions

AZI developed the system for information extraction and hypothesis generation and drafted the manuscript along with MS. MS also critically revised the manuscript for important intellectual content. DL supervised the work and gave final approval of the version of the manuscript to be submitted.

## References

[B1] Swanson DR Fish-oil, Raynaud's Syndrome, and undiscovered public knowledge. Perspectives in Biology and Medicine 1986 30 1 7 18 10.1353/pbm.1986.0087 3797213

[B2] Swanson DR Undiscovered public knowledge. Library Quarterly 1986 56 2 103 118 10.1086/601720

[B3] Swanson DR On the fragmentation of knowledge, the connection explosion and assembling other people's ideas. Bull. Amer. Soc. Inf. Sci. Technol 2001 27 3 12 14 10.1002/bult.196

[B4] Hu X Yoo I Song M Zhang Y Song I-Y Mining Undiscovered Public Knowledge from Complementary and Non-interactive Biomedical Literature through Semantic Pruning. ACM Fourteen Conference on Information and Knowledge Management (ACM CIKM 2005) 2005

[B5] Lindsay R.K Gordon M.D Literature-based discovery by lexical statistics. Journal of the American Society for Information Science 1999 50 7 574 587 10.1002/(SICI)1097-4571(1999)50:7<574::AID-ASI3>3.0.CO;2-Q

[B6] Pratt Wanda Yetisgen-Yildiz Meliha LitLinker: capturing connections across the biomedical literature. K-CAP'03 : Oct 2003 105 112

[B7] Srinivasan P Text mining: Generating hypotheses from MEDLINE. Journal of the American Society for Information Science 2004 55 4 396 413

[B8] Weeber M Vos R Klein H de Jong-Van den Berg L.T.W Aronson A Molema G Generating hypotheses by discovering implicit associations in the literature: A case report for new potential therapeutic uses for Thalidomide. Journal of the American Medical Informatics Association 2003 10 3 252 259 12626374 10.1197/jamia.M1158 PMC342048

[B9] Gordon MD Dumais S Using latent semantic indexing for literature based discovery. Journal of the American Society for Information Science 1998 49 674 685 10.1002/(SICI)1097-4571(199806)49:8<674::AID-ASI2>3.0.CO;2-T

[B10] Gordon MD Lindsay RK Toward discovery support systems: a replication, re-examination and extension of Swanson’s work on literature-based discovery of a connection between Raynaud’s and fish oil. Journal of the American Society for Information Science 1996 47 116 128 10.1002/(SICI)1097-4571(199602)47:2<116::AID-ASI3>3.0.CO;2-1

[B11] Lindsay RK Gordon MD Literature-based discovery by lexical statistics. Journal of the American Society for Information Science 1999 50 574 587 10.1002/(SICI)1097-4571(1999)50:7<574::AID-ASI3>3.0.CO;2-Q

[B12] Hristovski D Stare J Peterlin B Dzeroski S Supporting discovery in medicine by association rule mining in Medline and UMLS. Medinfo 2001 10 Pt 2 1344 8 11604946

[B13] Atkinson R Rivas A Discovering Novel Causal Patterns from Biomedical Natural-Language Texts using Bayesian Nets. IEEE Transactions on Information technology in Biomedicine 2008 12 6 10.1109/TITB.2008.920793 19000950

[B14] Srinivasan P Text mining: Generating hypotheses from MEDLINE. Journal of the American Society for Information Science 2004 55 4 396 413

[B15] Agrawal R U. Fayyad, et al Fast Discovery of Association Rules, Advances in Knowledge Discovery and Data Mining. 1995 AAAI/MIT Press

[B16] Srinivasan Padmini Thomas C. Rindflesch Exploring text mining from MEDLINE. Proceedings of the 2002 AMIA Annual Symposium 2002 2002 722 6 PMC2244226 12463919

[B17] Rindflesch TC Fiszman M The Interaction of Domain Knowledge and Linguistic Structure in Natural Language Processing: Interpreting Hypernymic Propositions. Biomedical Text Journal of Biomedical Informatics 2003 36 6 462 77 10.1016/j.jbi.2003.11.003 14759819

[B18] Fiszman Marcelo Thomas C. Rindflesch Halil Kilicoglu Integrating a hypernymic proposition interpreter into a semantic processor for biomedical text. Proceedings of the 2003 AMIA Annual Symposium 2003 PMC1479962 14728170

[B19] Porter MF An Algorithm for Suffix Stripping. Program 1980 14 3 130 137

[B20] Small Molecule Subgraph Detector http://www.ebi.ac.uk/thornton-srv/software/SMSD/

[B21] Dae-Hee Lee Juong G Rhee Yong J Lee Reactive oxygen species up-regulate p53 and Puma; a possible mechanism for apoptosis during combined treatment with TRAIL and wogonin. British Journal of Pharmacology 2009 10.1111/j.1476-5381.2009.00245.x PMC2743838 19438509

[B22] Mao Li Zhuo Zhang Hill Donald L. Xinbin Chen Hui Wang Ruiwen Zhang Genistein, a Dietary Isoflavone, Down-Regulates the MDM2 Oncogene at Both Transcriptional and Posttranslational Levels. Cancer Research 2005 65 18 16166295 10.1158/0008-5472.CAN-05-1302

